# A Framework for Effective Use of Hydroclimate Models in Climate-Change Adaptation Planning for Managed Habitats with Limited Hydrologic Response Data

**DOI:** 10.1007/s00267-015-0569-y

**Published:** 2015-07-04

**Authors:** Rachel A. Esralew, Lorraine Flint, James H. Thorne, Ryan Boynton, Alan Flint

**Affiliations:** U.S. Fish and Wildlife Service, Pacific Southwest Region Refuges Inventory and Monitoring Initiative, 3020 State University Drive East Suite 2007, Sacramento, CA 95819 USA; U.S. Geological Survey, California Water Science Center, Placer Hall, 6000 J Street, Sacramento, CA 95819 USA; Information Center for the Environment, Department of Environmental Science and Policy, University of California – Davis, One Shields Avenue, Davis, CA 95616 USA

**Keywords:** Climate change, Hydrologic model, Water supply, Managed wetland, Vulnerability assessment, Adaptation planning

## Abstract

Climate-change adaptation planning for managed wetlands is challenging under uncertain futures when the impact of historic climate variability on wetland response is unquantified. We assessed vulnerability of Modoc National Wildlife Refuge (MNWR) through use of the Basin Characterization Model (BCM) landscape hydrology model, and six global climate models, representing projected wetter and drier conditions. We further developed a conceptual model that provides greater value for water managers by incorporating the BCM outputs into a conceptual framework that links modeled parameters to refuge management outcomes. This framework was used to identify landscape hydrology parameters that reflect refuge sensitivity to changes in (1) climatic water deficit (CWD) and recharge, and (2) the magnitude, timing, and frequency of water inputs. BCM outputs were developed for 1981–2100 to assess changes and forecast the probability of experiencing wet and dry water year types that have historically resulted in challenging conditions for refuge habitat management. We used a Yule’s Q skill score to estimate the probability of modeled discharge that best represents historic water year types. CWD increased in all models across 72.3–100 % of the water supply basin by 2100. Earlier timing in discharge, greater cool season discharge, and lesser irrigation season water supply were predicted by most models. Under the worst-case scenario, moderately dry years increased from 10–20 to 40–60 % by 2100. MNWR could adapt by storing additional water during the cool season for later use and prioritizing irrigation of habitats during dry years.

**Electronic supplementary material:**

The online version of this article (doi:10.1007/s00267-015-0569-y) contains supplementary material, which is available to authorized users.

## Introduction

Increasing demand for limited water supplies of adequate quality to support the National Wildlife Refuge (refuge) ecosystems and management objectives is a growing risk for many refuges across the U.S., and is heightened during droughts and in the face of climate change. A major challenge for the U.S. Fish and Wildlife Service (USFWS) in light of this growing competition for water is to ensure that sufficient quantities of good quality water are available for fish, wildlife, and plants. Global hydroclimatic alteration is likely to exacerbate the scarcity of water resources for refuges, especially in California. For example, hydroclimatic projections indicate rising air temperatures from about 2–5 °C (Cayan et al. 2008), and higher spring and winter temperatures might result in earlier snowmelt runoff and a reduction in late spring and summer streamflow (Cayan et al. 2001; Mote et al. 2005; Stewart et al. 2005). Incorporation of predicted climate-change impacts to species- and land-management plans, programs, and actions, is needed to better understand potential underlying constraints to meeting current and planned habitat management objectives (USFWS 2010; Baxter et al.  2006).

Climate-change impacts to water availability are an important consideration when addressing limiting factors for the delivery of conservation obligations at the National Wildlife Refuges, as well as a critical step for development of species and habitat management plans and decision support tools. For example, climate change may result in substantial increases in constraints (temporal or quantitative) to established water rights and water supply scenarios (Parry et al. 2007, Medellin-Azuara et al. 2008, Hanak et al. 2011) on which conservation lands may be dependent (Pringle 2001). Water inputs to managed wetland systems provide resources for development, restoration, and maintenance of habitats. Delivered water is especially important or prevalent in managed wetlands in the western US that are converted from former agricultural lands with previously existing water rights and irrigation systems, and that often rely on diverted water (Pringle 2000; Fischman 2003). For example, 77 % of refuges in the Pacific southwest region of the US depend on diverted water (USFWS 2014). However, the impacts of climate change on the management of wetlands that are dependent on diverted water are not well discussed in the literature. A shift in the water regimes that can be expected as a result of climate change can ultimately affect the efficiency and sustainability of current or planned wetland management systems within a refuge.

In addition, much readily available information pertaining to the predicted effects of climate change on water availability is too broad in spatial scope or lacks enough specific information to understand impacts at the refuge scale. A local assessment, or “bottom-up” approach (Glick et al. 2011), can be used to address the exposure, sensitivity, and vulnerability of a refuge using known conservation goals and objectives. For example, the bottom-up approach can be useful for quantifying how climate change might change limiting factors of current and future habitat management for refuges that rely on diverted water for habitat management. The use of finer-scale climate-change modeling can improve our understanding of how much climate change affect specific water supply basins for a refuge, including modeling changes in the quantity, frequency, and timing of water delivery to the refuge. Furthermore, climate-change modeling can be used to estimate changes in the water balance within a refuge.

One modeling tool that can be helpful for evaluating the hydrologic response to climate is the Basin Characterization Model (BCM; Flint et al. 2013). BCM is driven by high-resolution (270 meter) downscaled precipitation and temperature data that are used to characterize the water balance at the land surface. The model can use either historical climate or future climate data, and downscaling from coarse grids to the 270-m spatial resolution is done for model application (Flint and Flint 2012; Flint et al. 2013; Flint and Flint 2014). Calculation of variables associated with the water balance incorporates static inputs (elevation, bedrock properties, soil properties), and time variable inputs (precipitation, temperature, and potential evapotranspiration derived from solar radiation) to produce water balance outputs (snow water equivalent, actual evapotranspiration, soil moisture, climatic water deficit (CWD), runoff, and recharge) for current conditions and forecasted for a range of climate-change scenarios on monthly and yearly time steps (Thorne et al. 2012).

Even though downscaled hydroclimatic data may be available for climate-change exposure assessments, at refuges where little quantitative information exists on thresholds of water supply that pose a threat to refuge sustainability, further approaches are needed. This is complicated for refuges with complex water delivery and management systems, complicated legal water allocation systems, and refuge management staff who have not quantified the impacts to habitat conservation from different quantities of water supply. A conceptual framework is needed to identify those hydrologic variables that are of most relevance to the way that refuges use and manage water.

The magnitude and frequency of extreme climate events, such as floods and droughts, can present challenges to management of water at refuges in which the degree of change that the refuge can adapt to is not quantified. Alternative qualitative tools can be used to help determine thresholds of high and low flow that might result in refuge management “stress,” such as interviews with refuge staff and reference of historic reports to determine resultant impacts to the refuge during wet or dry years, as defined by the quantity of discharge in water supply basins. Water year type designations (e.g., moderately wet year, extremely wet year) are commonly used to help define responses in water management practices to high and low flow conditions (Null and Viers 2013; Redmond 2002). In these frameworks, climate-change projections in combination with hydrologic response models such as the BCM can be used to forecast the frequency of occurrence of different water year types. Water year type frameworks can be useful for incorporation of climate change into the perspective of management outcomes and to enhance development of realistic and relevant adaptation strategies. Water year type frameworks can also be used to overcome a lack of site-specific information regarding the impact of different water supply regimes and can reduce the need for expensive and time-consuming secondary water-habitat-response models. Water year type frameworks might be cost-effective tools to apply not only to refuges and other conservation lands, but also to other water management systems such as those used for industrial, agricultural, and urban settings.

The Modoc National Wildlife Refuge (MNWR) was selected to test how the BCM can be used in evaluating the vulnerability of water management systems to climate change. The MNWR was selected because the refuge is highly dependent on diverted water to manage wetland habitats, and it is located in an area where water supply is geographically connected to source waters that are highly affected by snowmelt, which is estimated to be a direct driver of water supply stability (timing, magnitude, and frequency; Esralew et al. 2013).

The following questions were addressed for MNWR: (1) how much will climate change affect the general water balance properties within the refuge?: (2) how much will climate change affect the number and frequency of extreme events, and (3) how much will climate change affect the delivery timing of water in streams that supply water for MNWR? These data were used to address the implications of climate change for wetland and habitat management at the refuge and identify the potential adaptive capacity of the refuge to respond to changes in hydrologic response that would mostly impact on refuge habitat management.

To answer these questions, we demonstrate how use of a conceptual framework prior to use of downscaled climate models such as BCM can be an effective tool in focusing analysis on those hydrologic values that are hypothesized to have the most impact on refuge water use and management. We also demonstrate how use of a water year type framework that is qualitatively based on refuge accounts can help to effectively evaluate how climate change might affect the magnitude and frequency of extreme events, where quantitative thresholds for refuge adaptation capacity to climate change have not yet been established.

### Description of Study Area

**Fig. 1:**
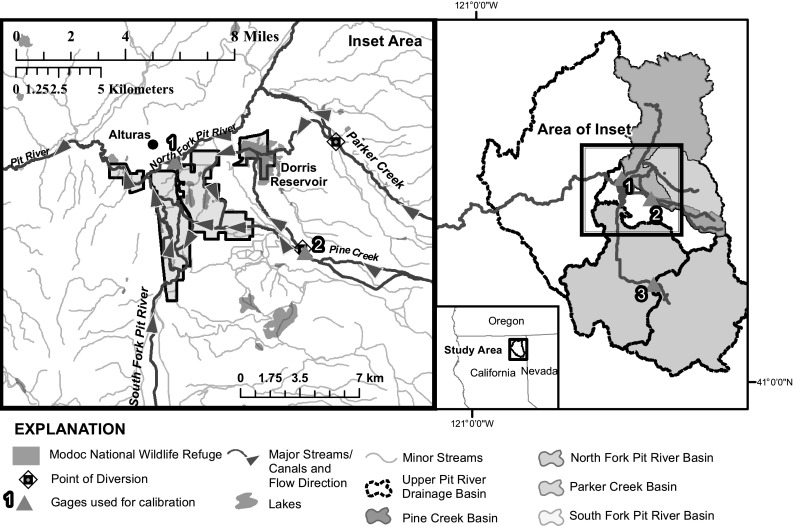
Surface water drainage basins and gages considered in study, and generalized flow system relevant for Modoc National Wildlife Refuge near Alturas California

The USFWS manages the 7021 acre MNWR in northeastern California near the confluence of the North and South Forks of the Pit River (Fig. 1) in the Upper Pit River Basin. The Upper Pit River Basin is a runoff dominated basin with substantial snowmelt from the Warner Mountains to the east (USFWS 2009; Esralew et al. 2013).

The primary purposes for the MNWR are to provide sanctuary for migratory birds; to provide habitat suitable for fish and wildlife-oriented recreational development; to develop, advance, conserve, and protect fish, and wildlife habitat; to manage other natural resources; and to conserve endangered species (USFWS 2009). MNWR meets its habitat management objectives through a complex and highly managed network of diversions that convey water from source tributaries to habitat management units. Water levels in the ponds and wetland units are manually managed to benefit waterfowl breeding success. Habitats that receive diverted water include semi-permanent, permanent, and seasonal wetlands, wet meadows, riparian areas, and croplands for wildlife food supply (Esralew et al. 2013).

MNWR diverts water directly from tributaries to the Pit River per terms of a complex series of water rights and decrees (Esralew et al. 2013). These tributaries include South Fork Pit River, Parker Creek, and Pine Creek (Fig. 1). Parker and Pine Creeks, and other smaller tributaries, contribute flow to the Dorris Reservoir from October to March (referred to as the “cool season”). The cool season also corresponds with California’s Mediterranean climate and is when most of MNWR’s annual precipitation falls (Esralew et al. 2013). Water in Dorris Reservoir is then re-diverted to the refuge during April-September (referred to as the “irrigation season”). Because of the proximity of Dorris Reservoir to Alturas, the reservoir also poses risks of flooding during extreme runoff years. The local community has come to expect the reservoir to be used to provide flood relief (S. Clay, USFWS, *personal communication*).

Water rights for North Fork Pit River are currently only exercised as diversions from Parker Creek (a tributary to North Fork Pit River) due to infrastructure limitations (S. Clay, USFWS, *personal communication*). However, flow in North Fork Pit River determines the legal ability of the refuge to divert water from both Parker and Pine Creeks, and is therefore important to refuge management.

The refuge also has four irrigation wells with pumps to utilize groundwater supplies of the Alturas Groundwater Basin to support habitat maintenance during extremely dry years. The refuge considers groundwater as an alternative supply to surface water that might be unavailable during drought conditions. Information about the specific quantity and frequency of groundwater use at the refuge was unavailable (Esralew et al. 2013).

A review of climate projections indicated that temperature was projected to increase while precipitation either increases or decreases depending on model scenarios (Esralew et al. 2013). Increased snowmelt and earlier runoff timing may increase the risk of flooding of Dorris Reservoir in certain years and decrease water supply later in the irrigation season when it is needed by the refuge for direct diversions as well as neighboring water uses. Increases in temperatures may result in an increase in water demands to maintain current habitats and decrease recharge thereby decreasing groundwater availability during times when MNWR needs alternative water sources to offset increases in irrigation demand (Esralew et al. 2013).

## Methods

### Use of a Conceptual Framework to Focus Vulnerability Analysis

**Fig. 2:**
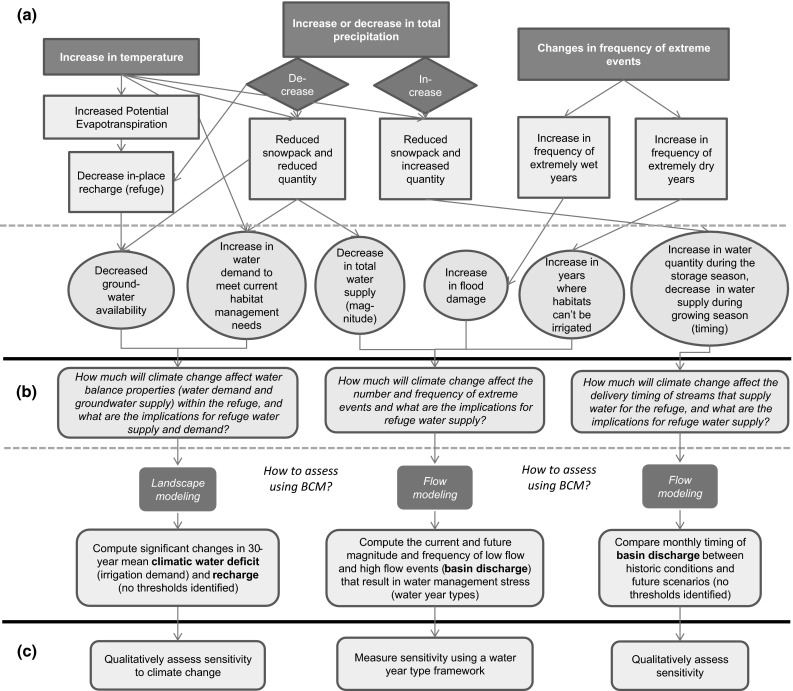
Conceptual framework describing a exposure with climate change, and hypothesized hydrologic response, b analysis questions and selected methods used to assess sensitivity of the water supply system at Modoc National Wildlife Refuge to climate change, and c qualitative or quantitative analysis of sensitivity of the refuge to changes in hydrologic response used to determine adaptive capacity

We first developed a conceptual framework to identify all possible changes, based on presumed certainties in climatic drivers (Fig. 2a). This conceptual framework was used to apply hypothesized changes in water resources at MNWR in order to identify hydrologic variables generated by BCM that would most directly highlight impacts to refuge water management. The conceptual framework is divided into three components: exposure, sensitivity questions and analysis methods, and assessment of sensitivity. Evaluation of exposure looks at potential hydrologic response in terms of refuge management, from increases in temperature and an increase or decrease in precipitation (Fig. 2a). Methods to assess sensitivity are identified for BCM to help measure the degree to which MNWR is sensitive to hypothesized hydrologic response to climate change (Fig. 2b). Based on the framework, chosen methods included analysis of CWD, recharge, and basin discharge. These hydrologic variables represent projected changes on both the landscape and in basin discharge (Fig. 2b). Sensitivity is assessed using resultant data either quantitatively or qualitatively (Fig. 2c). Adaptation capacity is determined qualitatively from results of sensitivity analysis.

To effectively measure the existing sensitivity, adaptive capacity, and vulnerability of wetland water management at MNWR to withstand climate change, thresholds of allowable alteration are required. Previously, no quantitative thresholds had been identified for how much landscape alteration or changes in discharge the refuge can withstand before experiencing negative consequences for management. MNWR also had limited information regarding managed wetland response to existing climate variability; only qualitative reports on wetland and habitat response were available.

To make best use of limited wetland response information, we developed a water year type framework to assess adaptive capacity, whereby we identify water year types in terms of qualitatively reported refuge management stress related to extreme and moderate high and low discharge events (Fig. 2c). We then used BCM-generated discharge for refuge water supply basins as a predictor of water year types, and used BCM to forecast discharge for these basins under future climate-change scenarios to estimate the relative frequency of these year types. By forecasting future water year types, we were able to forecast the frequency of years in which the refuge might be challenged to manage wetlands and other habitats under current operations and better assess the existing adaptive capacity of the refuge.

### Future Climate Scenarios

Global climate models (GCMs) are available for the continental United States at a 2.5 × 2.5 degree spatial resolution (Solomon et al. 2007; Parry et al. 2007). These projections have been downscaled to 1/8 × 1/8 degree (approximately 12 × 12 km [km]) spatial resolution using the constructed analogs method of Hidalgo et al. (2008) and the bias-corrected statistical downscaling (BCSD) method of Wood et al. (2004). These two methods are described and evaluated by Maurer and Hidalgo (2008). The projections developed using constructed analogs were statistically bias-corrected following Flint and Flint (2012).

We applied projections using medium to high CO_2_ emissions reflected in the A2 emissions scenarios (special report on emissions scenarios, Solomon et al. 2007) and RCP 6.0 (representative concentration pathways, Fujino et al. 2006).

**Fig. 3:**
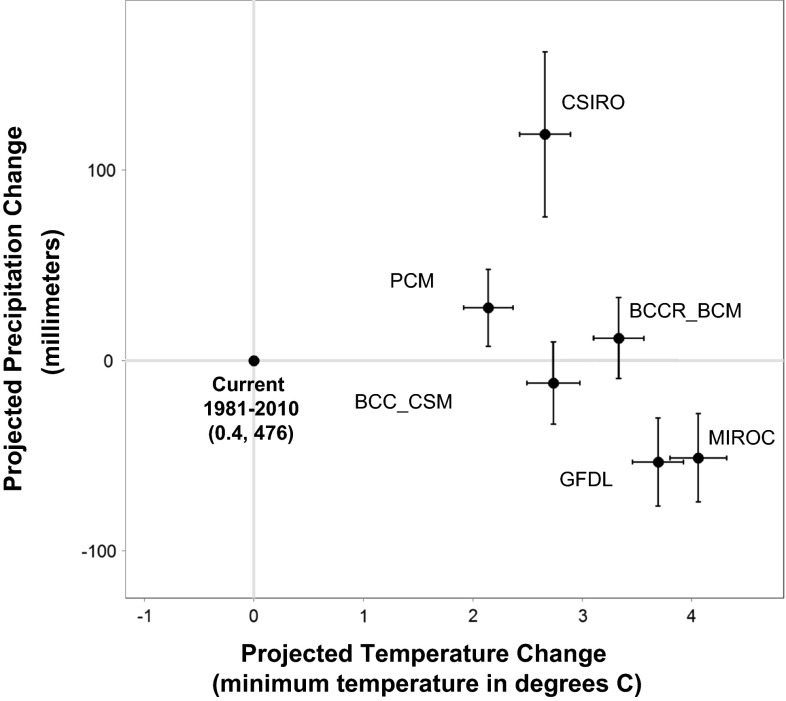
Changes in 30-year mean precipitation and temperature for an ensemble of six climate model projections over the Upper Pit River Basin, 2070–2099. All models are considered for the A2 greenhouse gas and emissions scenario except BCC_CSM which is considered under the RCP6.0 scenario

We selected futures for the MNWR application to represent a range of projected precipitation and air temperature conditions spanning from warm and wet to hot and dry. We selected six models with varying levels of change in precipitation and temperature from historical to potential future conditions, with the purpose of selecting distinct climate scenarios (Fig. 3). Details about each selected model are provided in supplementary materials. While updated IPCC climate projections have been issued since this study was conducted, our stratifying of climate futures into wet and dry conditions still represents a suitable set of alternatives to use for scenario planning purposes.

The six projections used in our study were spatially downscaled from the 12-km grid scale to 270-m for model application (Fig. 3) using the Gradient-Inverse Distance Squared (GIDS) spatial interpolation approach described in Flint and Flint (2012).

### Description of the Basin Characterization Model (BCM)

**Fig. 4:**
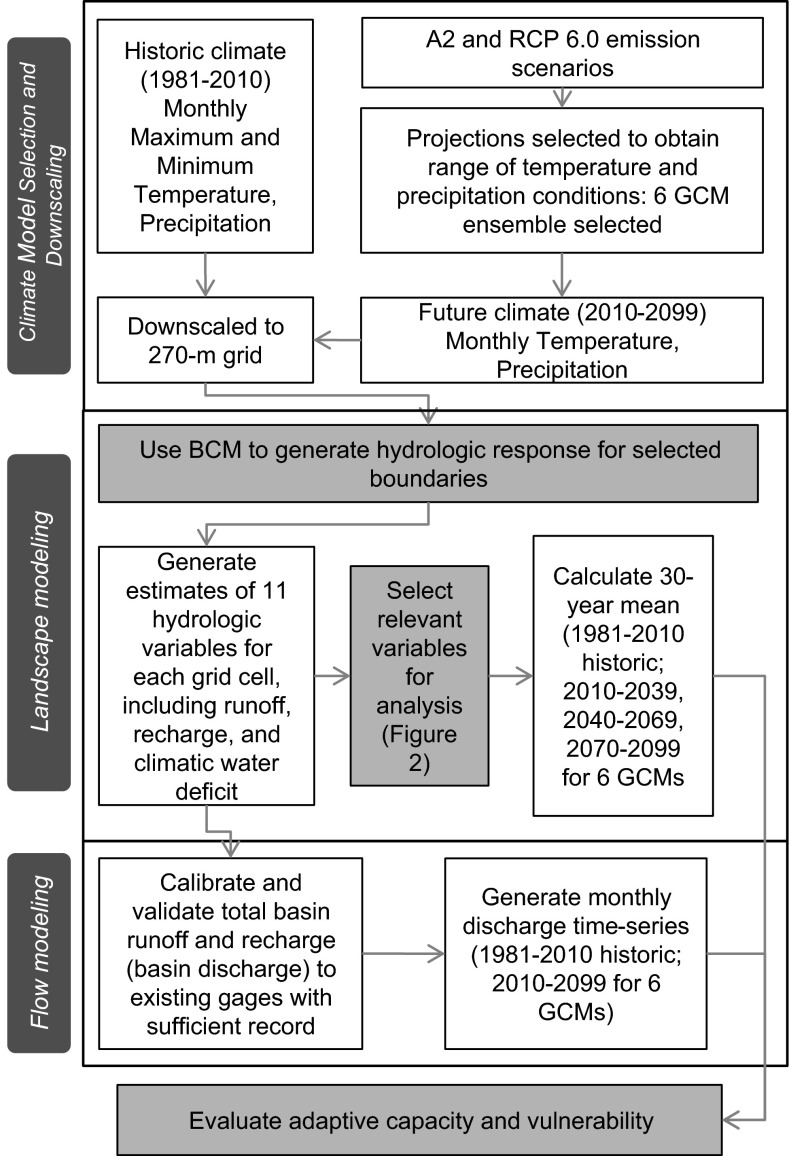
Conceptual schematic of methods used to downscale climate information and generate variables used to evaluate adaptive capacity and vulnerability of water management at Modoc National Wildlife Refuge to climate change

The BCM mechanistically models the pathways of precipitation into snow, evapotranspiration, soil infiltration, runoff, or recharge (Flint et al. 2013). The BCM can be used to generate up to 14 hydrologic variables, but a smaller set of variables was used to focus on the water balance properties that are most relevant to the way that MNWR manages and uses water (Fig. 2). Historical precipitation and temperature data used in this study was based on 800 meter PRISM raster grids (Daly et al. 2008) which were further downscaled to 270 meter using methods described in Flint et al. (2013). Snow water equivalent (based on snow depth, accumulation, and snowmelt) was calculated based on precipitation and temperature from downscaled PRISM using methods described in Flint and Flint (2007). Water content at field capacity and wilting point, porosity, and depth were derived from the SSURGO soil database (Natural Resources Conservation Service 2006). Potential evapotranspiration was calculated on the basis of solar radiation, slope and aspect, topographic shading, and atmospheric conditions. CWD is calculated as potential minus actual evapotranspiration and represents seasonal demand for water and landscape stress. The BCM calculates hydrologic variables on a grid cell basis developed at a resolution of 270 meters, which were summarized over selected delineated basins shown in Fig. 1. Basin discharge is calculated from recharge and runoff (Fig. 4) summed for all grid cells up stream of a stream gage, and post-processing is done to match the measured hydrograph for model calibration using assumptions of surface, shallow, and deep water reservoirs (Flint et al. 2013).

To evaluate the hydrologic response to climate for each selected basin that contributes water to MNWR, we used the BCM to calculate hydrologic conditions across the landscape for 1981–2010 and projected them for the six modeled scenarios for 2010–2099 (Fig. 4). Trends in climate, hydrologic derivatives of runoff and recharge, and CWD are separately analyzed for historic‐to‐future time periods (1981–2010 to 2070–2099).

### Selection of Spatial Boundary Conditions

We selected the Upper Pit River drainage basin as an overall boundary condition for BCM analysis (Fig. 1). The Upper Pit River drainage basin included smaller tributaries that completely encompassed the last point of drainage for MNWR and that were used in subsequent model calibration. Basin numbers are used to sum BCM outputs (Fig. 1; Table 1).

**Table 1:** Streamflow gaging stations used to calibrate basin characterization model within the Upper Pit River Basin

Map ID (Fig. 1)	Drainage basin area (km^2^)	Streamflow gaging station name	Source	Station identification number	Period of record
1	549.1	North Fork Pit River at Alturas, Calif.	USGS National Water Information System (NWIS, http://waterdata.usgs.gov/nwis)	11344000	1972–1985
2	61.9	Pine Creek State Gage	California Department of Water Resources Water Data Library (http://www.water.ca.gov/waterdatalibrary/)	A14100	1975–1996
			USFWS WISKI Database (S. Fluter, U.S. Fish and Wildlife Service, *personal communication*, Aug. 2012)	169017	2005–2012
3	639.7	South Fork Pit River near Likely, Calif.	USGS NWIS	11345500	1928–2012

We evaluated time-series trends in basin discharge in selected basins that contribute to refuge water inflow (Figs. 1, 4). These basins include South Fork Pit River upstream of the refuge boundary, Pine Creek upstream of all diversions, Parker Creek above the confluence with the North Fork Pit River, and North Fork Pit River above its confluence with the Pit River (Fig. 1).

An area of overland runoff, a small portion of which drains to Dorris Reservoir, was not included in this analysis (white area shown between basins in Fig. 1). Some water is diverted for neighboring land-use although the fraction of water from this basin that is contributed to Dorris Reservoir and the refuge is unknown. Furthermore, this area is assumed to be a minor source of supply because this area only contributes about 15 % of the total water supply to Dorris Reservoir (Esralew et al. 2013).

### Model Performance: Calibration and Validation

BCM was calibrated to three gaging stations that were used in this study that best represented flow within the selected boundary (Table 1). The regional BCM, developed for California, was applied to the study area following regional calibrations for solar radiation, PET, and snow cover, along with local calibrations to several hundred streamflow gages (Flint et al. 2013). Because sufficient gage record was unavailable for Parker Creek, we applied the calibration coefficients from the neighboring basin of Pine Creek, of similar size, and assumed that the geology and land use between these two basins were similar. Pine Creek State Gage (station 2) was operated by two different agencies over the period of record, but the gage location did not change (Table 1).

Calibration of BCM output is achieved by applying exponent coefficients to the BCM output to improve baseflow estimates (Flint et al. 2012). Because the monthly water balance of each grid cell is not connected to upstream or downstream cells and does not have carryover, zero flows commonly occur during seasonal and annual dry periods. We transformed BCM through calibration using empirical flow-routing equations into a form that can be compared to the pattern and quantity of measured streamflow at gages. The water balance was conceptualized as consisting of runoff and recharge that occur within three distinct reservoirs with surface flow, shallow flow, and deep flow, each with exponents (ranging from 0 to 1) that describe recession in different parts of the streamflow hydrograph. Greater exponent coefficients increase the amount of water that is carried to the next month as baseflow. Values are adjusted manually until pattern, and quantity of discharge at the gaging stations is best matched. Further details of equations used are in Flint et al. (2012).

All of the available streamflow from gages in the study area was affected by some form of regulation as a result of reservoir operations and upstream diversions for irrigation and water for livestock. Exponent coefficients were modified in the calibration equations to maintain mass balance between the measured streamflow and simulated streamflow by limiting the contribution of the shallow groundwater reservoir to streamflow.

The most regulated reaches were South Fork Pit River and North Fork Pit River. The West Valley Reservoir is just above the gage on South Fork Pit River and is used to flood irrigate wild rice upstream from the refuge; but during wet years, excess water in the reservoir is sold to downstream irrigation districts (Esralew et al. 2013) and is therefore not accounted for at the gaging station. The gage at North Fork Pit River is downstream of numerous diversions for irrigation; therefore, this water is not accounted for at the gaging station. Information about the response of diversions and reservoir operations to changes in water availability were not available for analysis. Exponent coefficients were used to simulate removal of water from streamflow that would be regulated or diverted, proportional to the modeled streamflow in the system. For purposes of this study, an assumption was made that these calibration coefficients remain constant in future climate-change forecasts (for example, indicating that reservoir practices remain constant). However, if diversions or reservoir practices change in the future, the coefficients used in this analysis may no longer be relevant.

### Analyses of Selected Climatic and Hydrologic Variables

We used the conceptual framework (Fig. 2) to identify all possible changes based on presumed certainties in drivers and identified three important indicator metrics (CWD, recharge, and basin discharge) to represent projected changes on the landscape by evaluating changes in 30-year summaries and in basin discharge by evaluating changes in time series for the Upper Pit River Basin.

To assess the sensitivity of the refuge to these changes, we assessed groundwater availability and wetland water demand by analyzing 30-year average annual mean values of CWD and recharge within the refuge boundary and in the Upper Pit River Basin (Figs. 2c, 4). In-place recharge is actually an underestimate of actual recharge within the refuge boundary since this modeled parameter does not take into account external irrigation water for wetland management (which would result in artificial recharge), but rather, is an indicator of changes in recharge relative to in-place precipitation within the refuge boundary.

To determine the impact of climate change on refuge water supply, we generated monthly time series of runoff and recharge estimates (Figs. 2, 4). We used these to assess shifts in total basin yield for four analysis sub-basins, the North Fork Pit River, Parker Creek, Pine Creek, and South Fork Pit River, with relevance to water supply at MNWR and to examine the frequency of exceedance of extreme high and low flow values (Fig. 1). South Fork Pit River was estimated as the discharge at the outlet of the South Fork Pit River Basin at the refuge boundary, including water that may be diverted upstream for use on agricultural lands, but which may eventually drain back to the river.

#### 30-Year Summaries by Refuge Boundary and Water Supply Basin

For the MNWR Basin, we used the BCM to produce 270 meter grids to represent historic and future climates for the variables described above. The mean and standard deviation of annual (water year) values were computed over a 30-year period for the following time periods: 1981–2010 and future time slices (2010–2039, 2040–2069, and 2070–2099).

We examined patterns in CWD and recharge by computing statistical changes in these parameters within the refuge boundary and Upper Pit River Basin (water supply boundary). We compared the statistical significance of temporal changes in 30-year mean CWD and recharge using a basic Student’s *P* test on each pixel within the water supply boundary. The t-tests were performed between the 30-year mean of the historic period and the 30-year mean of all three future time periods for each model. A de-trended standard deviation was used to determine if variability in the 30-year mean was present in the absence of a persistent trend over time. The de-trended standard deviation was computed as the residual on a linear regression between values over the 30-year period. We estimated the proportion of area within the refuge boundary and water supply boundary that we predicted to experience significant changes in each hydrologic variable by computing the percentage of each area in which grid cells showed significant changes by the t-test.

#### Time-Series Analysis for Selected Basins

To investigate how climate change might impact the frequency of exceedance of extreme events, we analyzed changes in the frequency of annual and seasonal streamflow thresholds for high and low flow for each sub-basin. Initially, data about the thresholds of concern for annual refuge inflow that historically resulted in refuge stress were unavailable, and cutoff selection was complicated by the complex water right allocation system. Therefore, we used several historical sources of qualitative information to characterize refuge vulnerability to extreme high and low flow thresholds (referred to as “water year types”), including MNWR Annual Narratives and Water Management Plans (https://ecos.fws.gov/ServCat/Reference/), monthly Dorris Reservoir water level information provided from refuge records (data source and availability, Esralew et al. 2013), and refuge staff interviews. The MNWR annual narratives were available from 1971 to 1990 and 2002–2005, and annual water management plans were available from 1971 to 1973, 1981 to 1990, and 1992. Monthly Dorris water level data were available from 1976 to 1992, 1994 to 1997, and 2000 to 2011. Refuge staff recalled experiences and responses since 1992.

Based on these sources, we identified five water classes and assigned each year to a type (Table 2). An assumption was made that current refuge staff had similar management responses (habitat management in dry years and flood management) as past staff, in terms of implications of extreme wet and dry conditions.

**Table 2:** Definitions of water year types as defined by refuge habitat management outcomes

Water year type	Refuge management definition	Years identified (calendar year) and source code
Extremely wet	Flooding resulted in damage to infrastructure resulting in significant repair costs	1971^a^, 1980^b^, 1986^a^, 1998^c^, 2006^c^
Moderately wet	Flooding resulted in staff time expended in flood prevention maintenance or resulted in temporary damage to wildlife habitat (such as nest or other habitat destruction, but which reflect periodic disturbance that might be experienced in a natural system)	1982^a^, 1983^a^, 1984^a^, 1993^c^, 1996^c^
Normal	Water supply was abundant or adequate to meet refuge habitat management demands	1972–1976^a^, 1978–1979^a^, 1981^a^, 1985^a^, 1987^a^, 1989^a^, 1991^a^, 1994–1995^a^, 1997^c^, 1999–2000^a,c^, 2002–2005^a,c^, 2008–2010^c^
Moderately dry	Habitat management and maintenance were prioritized based on available water; Dorris Reservoir did not fill to capacity (indicating that water availability was less than optimum)	1977^a,b^, 1988^a,b^, 1990^a,b^, 2000^b,c^, 2002^a,b,c^, 2007^b,c^
Extremely dry	Refuge staff were unable to adequately meet planned annual habitat objectives; refuge relied heavily on groundwater pumping to account for lack of surface water supply	1987^a,b^, 1992^b,c^, 2001^b,c^

^a^Modoc National Wildlife Refuge Annual Narrative and/or Annual Water Management Plans

^b^Dorris water level records (Modoc National Wildlife Refuge digital communication, October 2012)

^c^Modoc National Wildlife Refuge management staff, oral communication, April 2013

For 1971–2010, we estimated the exceedance percentile of modeled annual or seasonal streamflow that most accurately identified extremely wet and extremely dry years (Table 2). Because streamflow in each sub-basin may respond differently to annual climate conditions and annual climate conditions may be different among basins, we tested the best aggregation of flow data by computing total modeled monthly discharge for each of the four sub-basins as annual and seasonal sums, and computed 2-year annual averages. All annual sums and averages were by water year (October through September). Percentiles tested included 95, 90, 85, 80, and 75th exceedance percentiles for low flow and 5, 10, 15, 20, and 25th exceedance percentiles for high flow. Modeled flow was not tested for seasonal statistics for South Fork Pit River because upstream reservoir regulation likely impacted accurate seasonal computations of modeled flow; actual discharge was less than modeled discharge during the cool season as a result of water storage and greater than modeled discharge during the irrigation season during summer releases.

We determined the combination of percentile and annual and seasonal statistics that most accurately identified extremely wet, moderately wet, moderately dry, and extremely dry years for each of the four basins, as indicated by a maximized Yule’s Q skill score (Yule 1900, Agresti 1996), here called “Yule’s Q.” Yule’s Q uses a contingency table to compute the ratio of the odds of making a successful prediction given that the event occurred (i.e., a “hit”) to the odds of making an unsuccessful prediction given that the event failed to occur (i.e., a “false alarm”; Stephenson 2000). Yule’s Q ranges from 0 to 1, where 1 indicates that all predictions were successful, and 0 indicates than no successful predictions were observed (Yule 1900). Yule’s Q is a measure of model efficiency in binomial result cases because it is a single measure that summarizes the degrees of freedom in the conditional joint probability distribution (Stephenson 2000).

While Yule’s Q is useful for comparing general model efficiency among models, a Fisher’s exact test (Fisher 1970) can indicate whether the successful model predictions under any model were actually statistically significant. We computed *P*-values with Fisher’s exact test whereby the null hypothesis of no correlation was rejected at a *P*-value of 0.05. If any cell counts were equal to zero, the model was not considered in further testing. All statistical tests were performed in R using the “stats” library package. The odds ratio and standard error computations are described in Stephenson (2000).

We computed Yule’s Q for all models in which the probability of predicted exceedance was significantly greater than a score of zero at a confidence interval of 95 %. We then selected the model with the greatest value of Yule’s Q for each water supply basin. In the case of a tie, we made an attempt to select consistent percentiles and annual and seasonal statistics among basins. After consistency was attempted to resolve ties, annual aggregations were generally preferred over seasonal and moving averages. We then computed a final Yule’s Q for all selected basin models to compare accuracy of water year type prediction when at least one exceedance was detected among each of the four basin models for any given year. A final score for combined basin models was evaluated because refuge historic reports did not typically specify the likely cause (source basin) of wet or dry conditions. The Yule’s Q for combined basin models was compared to individual basin scores to determine if model skill was improved or harmed by predicting water year types by exceedance in any one of the four basins. We then selected the model (individual basin or combined basins) with the highest Yule’s Q skill score for each water year type.

## Results and Discussion of Model Simulations

### Model Calibration Results

**Fig. 5:**
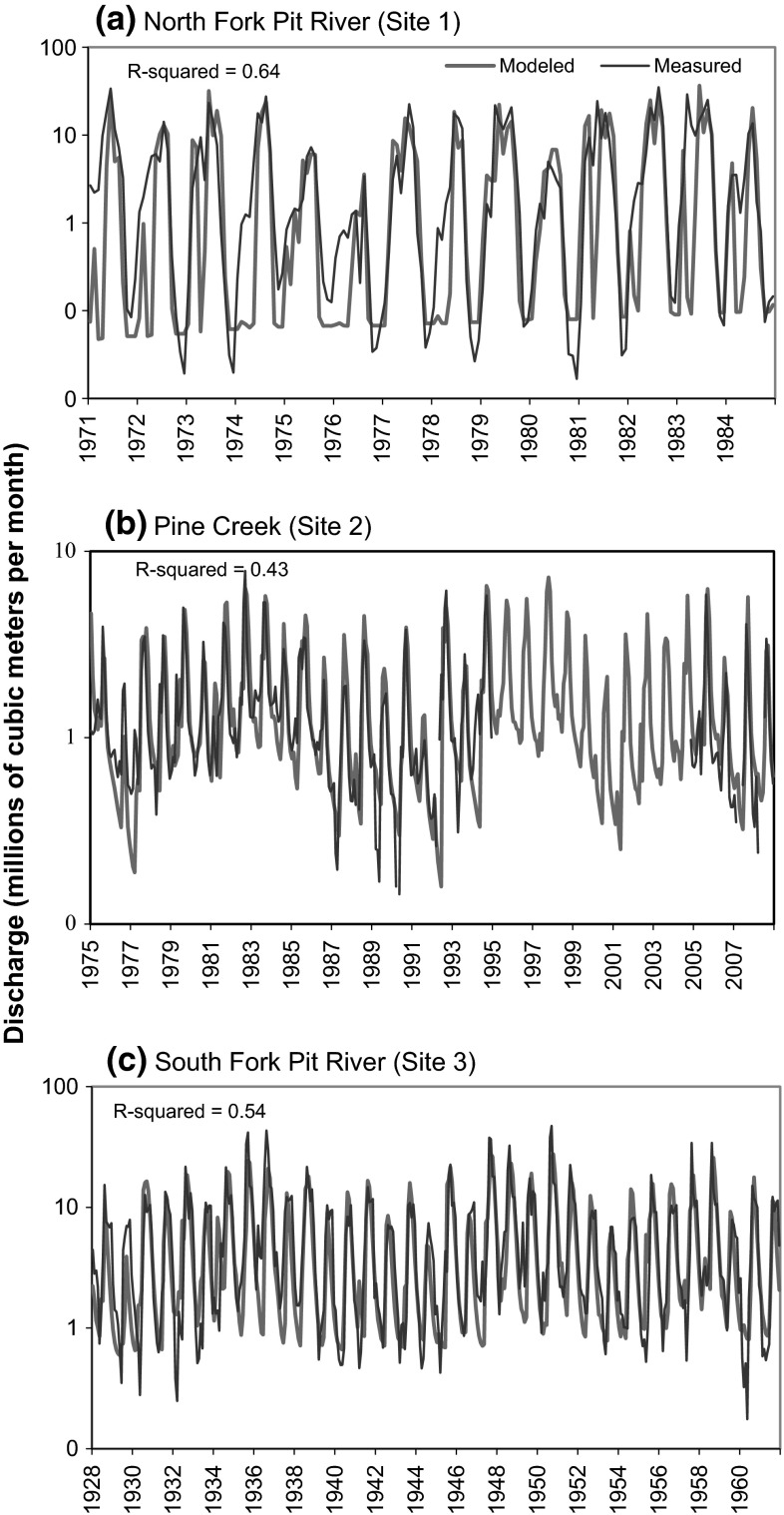
Calibration time series comparing measured and estimated basin discharge for **a** North Fork Pit River. **b** Pine Creek. **c** South Fork Pit River

Results from the BCM model calibration indicated poor-to-moderate fit between monthly modeled and measured discharge, with R-squared values 0.43, 0.54, and 0.64 (Fig. 5). All linear regressions were statistically significant at a *P*-value of less than 0.0001. Pine Creek had the poorest fit. Causes for poor model fit at the North Fork and South Fork Pit River include regulation of flow reflected in measured discharge. Poor model fit for all stations might have been caused by local volcanic geology comprised numerous faults and highly heterogeneous strata (Miles et al. 1997), which might have complicated applied recharge coefficients. However, the inter-annual timing and patterns of high and low flow were fairly well preserved in most years between modeled and measured discharge. Therefore, we assumed that use of the model would be sufficient to predict the frequency of refuge water year types.

### Changes in Water Supply Drivers and Refuge Water Characteristics

Although mean 30-year precipitation did not significantly change for most future projections in general, by the end of the century: non-significant decreases in the mean were observed for the GFDL and MIROC models; non-significant or significant increases were observed in the mean for the CSIRO model; and both non-significant decreases and increases were observed for the BCCR_BCM2 and BCC_CSM models depending on area (Table 3). We therefore used groupings of “drier models,” “wetter models,” and “precipitation-neutral models,” respectively, to investigate any patterns in results by model type.

**Table 3:** Changes in the 30-year mean of climatic water deficit and recharge from the Basin Characterization Model from historic (1981–2010) to 2070–2099 within the Modoc National Wildlife Refuge boundary and the surrounding water supply basin

Parameter	Selected Boundary	Statistic	Historic (1981–2010)	MIROC	GFDL	BCC_CSM	BCCR_BCM2	PCM	CSIRO
Climatic Water Deficit (mm)	Refuge boundary	Mean	584	773	760	699	702	672	628
		Percent dif.	NA	***32.4***	***30.1***	***19.7***	***20.2***	***15.1***	***7.5***
		Percent of sig. grid cells	NA	***100***	***100***	***100***	***100***	***100***	***28.4***
	Water supply basin	Mean	586	786	753	707	721	674	645
		Percent difference	NA	***34.1***	***28.5***	***20.8***	***23.1***	***15.1***	***10.1***
		Percent of sig. grid cells	NA	***100***	***100***	***100***	***100***	***99.8***	***72.3***
Recharge (mm)	Refuge boundary	Mean	15.3918	8.5	5.4	10.7	14.6	12.9	24.2
		Percent difference	NA	−***44.7***	−***64.9***	−***30.8***	−***5.5***	−***16.0***	**56.9**
		Percent of sig. grid cells		None	***44.2***	***<1***	None	None	None
	Water supply basin	Mean	46.5808	39.6	35.5	47.2	49.7	46.9	66.5
		Percent difference	NA	−***14.9***	−***23.84***	**1.34**	**6.78**	**0.58**	**42.8**
		Percent of sig. grid cells		*5.5* ^1^	**27.3**	*11.4* ^2^	**1.03**	**<1**	**32.9**

^1^1.06 % are significantly decreasing and 4.53 % are significantly increasing

^2^2.4 % are significantly decreasing and 8.96 % are significantly decreasing

Percent difference is between predicted future mean and historic mean; Percent of sig. grid cells is the percentage of grid cells in the selected boundary in which the predicted future 30-year mean was significantly different than the historic period at a 95 % confidence interval. ET is evapotranspiration. Bold-italicized text indicates drier or hotter conditions, or less water availability; bolded text without italics indicates wetter or cooler conditions, or more water availability; italics text without bold indicates mixed results

Within the refuge boundary, temporal trends in de-trended 30-year means of CWD indicated fairly consistent increases in CWD toward the end of the century. An exception was CSIRO model in which CWD only increased for about 28 % of the refuge (Table 3). The CSIRO model also predicted increases in recharge over about 57 % of the refuge (Table 3). The other models all predicted significant decreases in discharge on the refuge affecting 5.5–65 % of refuge area. .

Recharge increased with the CSIRO model by 42.8 in 33 % of the water supply basin, but no grid cells showed significant differences within the refuge boundary (Table 3). Recharge increased slightly (6.78 % or less) for the precipitation-neutral models BCC_CSM and BCCR_BCM2, and wetter PCM model, in the water supply basin. However, these increases were over a small area, with less than 10 % of the basin area affected. Recharge significantly decreased mostly for the GFDL model, with −23.8 % over 27.3 % of the water supply basin, and −64.9 % over 44.2 of the refuge boundary (Table 3). MIROC did not predict significant decreases within the refuge boundary and showed only slight decreases in the water supply basin (1 %).

### Changes in Water Supply Discharge

#### Timing and Magnitude of Discharge

**Fig. 6:**
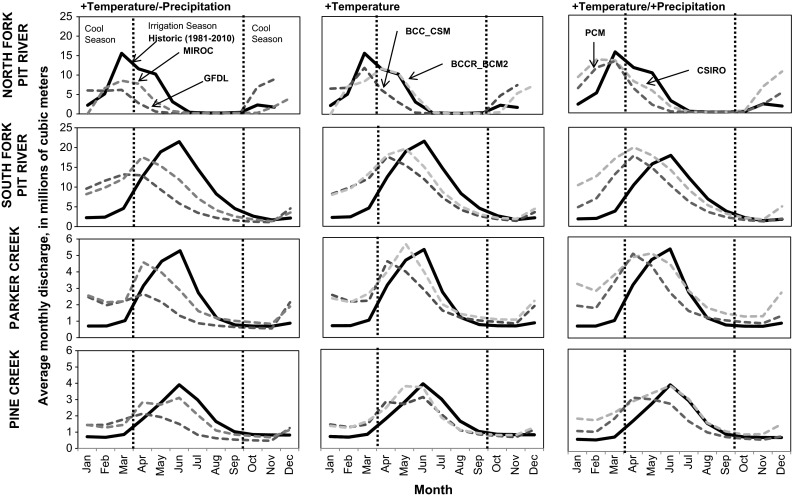
Mean 30-year monthly averages of discharge for the period 1981–2010 and six future projections for drier models, precipitation-neutral models, and wetter models, for 2070–2099 modeled for four contributing watersheds to Modoc National Wildlife Refuge 3.3.2 frequency of extreme events

30-year mean monthly hydrographs and time-series plots for modeled discharges for the period 2070–2099 show earlier timing of peak magnitude of monthly discharge compared to historic conditions for all models and basins with the exception of North Fork Pit River (Fig. 6). Timing of peak monthly discharge at North Fork Pit River was March under the historic models. Timing of peak monthly discharge at North Fork Pit River did not change under the MIROC, GFDL, and BCC_CSM models and was one monthly later under the BCCR_BCM2 model. For the other basins, forecasted peak magnitude generally occurred in April or May compared with an historic peak in June.

**Fig. 7:**
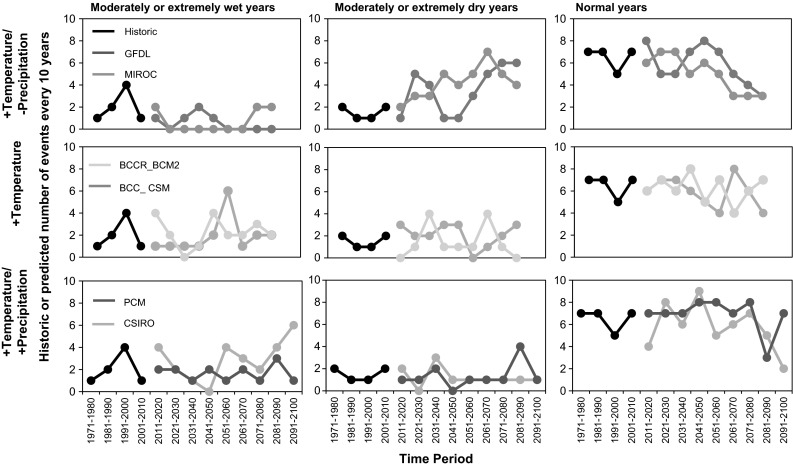
Historically recorded and projected 10-year frequency of exceedance of water year type thresholds for moderately or extremely dry years, moderately or extremely wet years, and normal years under drier climate models, precipitation-neutral climate models, and wetter climate models

All models showed a greater magnitude of cool season flows, with increases ranging from 7.1 to 270 % of historic cool season discharge among all basins (Fig. 6). Greater discharges are apparent for most models from November or December through April or May (Fig. 7).

Most models showed a decrease in the magnitude of irrigation season flows. However, the CSIRO and BCCR_BCM2 models show some increases in irrigation season discharge for some basins. The CSIRO model showed increases in irrigation season discharge of 4.1–14 % for all basins except the North Fork Pit River. The BCCR_BCM2 showed a small increase in irrigation season discharge in Parker Creek (2 %).

The results of the Yule’s Q test were variable among basins and likely reflect the variable flow characteristics of each water supply basin (Table 4). Yule’s Q values ranged from 0.92 to 0.98 for all selected thresholds.

**Table 4:** Selection of metrics used to estimate occurrence of water year types as defined by refuge habitat management outcomes

Refuge water year type definition	North Fork Pit River		Parker Creek		Pine Creek		South Fork Pit River		Combined Yule’s Q skill score	Final model (basin/percentile exceedance/statistic)
	Selected metric (percentile exceedance/statistic)	Yule’s Q skill score	Selected metric (percentile exceedance/statistic)	Yule’s Q skill score	Selected metric (percentile exceedance/statistic)	Yule’s Q skill score	Selected metric (percentile exceedance/statistic)	Yule’s Q skill score		
Extremely wet	10/Annual	0.92	20/Annual	0.92	20/Annual	0.94	20/Annual	0.923	Not computed	Not used
Moderately wet or extremely wet	15/Annual	0.93	20/Annual	0.93	20/Annual	0.98	20/Annual	0.95	0.95	Pine Creek/20/annual
Moderately dry or extremely dry	85/Irrigation season	0.95	85/Irrigation season	0.95	85/Irrigation season	0.95	85/Annual	0.95	0.86	Pine Creek/85/irrigation season
Extremely dry	80/Irrigation season	0.94	80/Irrigation season	0.94	90/Irrigation season	0.96	80/2-year moving average	0.94	Not computed	Not used

Irrigation season is April to September. North Fork Pit River, Parker Creek, Pine Creek, and South Fork Pit River are contributing watersheds to Modoc National Wildlife Refuge

Model results were occasionally illogical and may have resulted from lesser counts of false positives in moderate years compared to extreme years. For example, the 85th percentile of modeled irrigation season discharge in Parker Creek Basin was the best predictor of moderately dry years, whereas the 80th percentile of modeled discharge for the same season was the best predictor of extremely dry years. In some cases, the percentiles and annual and seasonal statistics were the same between extremely wet years and moderately wet years (Supplemental Table 2). We then determined that that percentile models were generally no better at predicting extreme years than moderate years and were therefore combined. The Pine Creek model was selected as an indicator of water year type because model skill was generally greatest among basins. Model skill was not improved by combining basins. Model skill for estimating moderately or extremely dry water year types was no different among any given basin (Yule’s *Q* = 0.95), but when combined, was substantially poorer (Yule’s *Q* = 0.86). Pine Creek model was selected to estimate all water year types for consistency, and because moderately or extremely wet and moderately or extremely dry years were always mutually exclusive (no year was both wet and dry). North Fork and Parker Creek showed some overlap in water year type detection. For example, up to 6.2 % of years for the North Fork, model indicated both wet and dry years. This could occur because the statistic used to estimate wet and dry years were different.

The frequency at which the discharge criteria in Table 4 were met in each decade for each modeled scenario is shown in Fig. 7. Normal years were also plotted and were defined as those years that were not predicted to be at least moderately wet or moderately dry. Exceedance of combined thresholds shows increases or decreases depending on model type. The wettest model (CSIRO) showed increases in the frequency of moderately wet or extremely wet years of up to 6 times per every 10 years by the end of the century, compared to about 1–3 times per every 10 years historically. The frequency of years that are at least moderately dry or extremely dry increased under the driest model scenarios, but did not change substantially under the other models. Under the driest scenarios, the frequency of years that were moderately dry or extremely dry will likely increase from the historic range of 10–20 % of the time to 40–70 % of the time by the end of the century. Both wetter models (CSIRO and PCM) showed decreases in the frequency of drier years.

## Conclusions

### Implications for Refuge Water Management

Addressing the security of future water supply for refuges can benefit long-term refuge management planning because alternative management options may be identified to help develop adaptation strategies to mitigate the detrimental effects of predicted changes on the refuge. For example, decisions can be made to increase water diversion and storage capacity to offset increases in irrigation demand, or to decrease the vulnerability of the refuge to detrimental flood damage through changes to planned habitat management. Where uncertainty of the effects of projected climate exist that might preclude development of clear adaptation strategies, steps can also be taken to improve monitoring to reduce uncertainty or potentially prepare for the range of possible outcomes using risk-averse approaches.

While GCMs generally agree on increasing air temperature projections for the MNWR region, these models indicate uncertainty in the direction of precipitation trends which was the greatest source of uncertainty in the resultant hydrologic processes that impact refuge water supply. Regardless of the direction of precipitation trends, CWD increased in all models, which indicates that greater irrigation will be needed to maintain current vegetation and habitats in the future. Recharge did not significantly increase within the refuge boundary under any model, and one dry model (GFDL) showed the greatest extent of significant reductions in recharge on and near MNWR. This indicates that groundwater may not be a reliable resource in the future as an alternative water supply source to meet these increased demands.

Earlier future discharge implies greater cool season discharge and lesser irrigation season discharge for South Fork Pit River, Pine Creek, and Parker Creek for most models. This could impact the ability of the refuge to directly divert water during the irrigation season. Dorris Reservoir can remain a valuable asset for mitigating the effects of reduced irrigation season discharge, as long as the reservoir remains at a large enough capacity to take advantage of increases in the availability of water in Pine and Parker Creek to store during the cool season.

Drier model scenarios reduce the refuge’s ability to divert surface water during the irrigation season while concurrently decreasing recharge, which may impact the refuge’s ability to pump groundwater to offset the lack of surface water supplies. Under the drier models, moderately dry years as indicated by water supply (years in which habitat management and maintenance practices were prioritized based on limited water availability) will likely increase from 10 to 20 % of the time historically to 40–60 % of the time by the end of the century, while dry years may remain the same under the precipitation-neutral and wetter scenarios. However, these estimates of frequency are an underestimate because increases in CWD will require more water to meet existing habitat demands and may result in less habitat management success with the same water deliveries in the future.

Under the wetter scenarios, frequency of drier years will likely not change and MNWR can adapt by utilizing groundwater during dry spells. However, increases in the frequency of wet years (as observed for the CSIRO model by the end of the century) and the magnitude of wet year inflows could increase the risk of damage to the refuge and to the nearby city of Alturas from flooding.

MNWR could consider development of adaptation strategies to mitigate these potential changes in spite of model uncertainty. Increasing the ability to store water in the cool season in Dorris Reservoir (through dredging or reservoir expansion) would increase the refuge’s capacity to deliver additional water to habitat units in the irrigation season when more water is needed, especially under drier scenarios, and better buffer against flood damages under wetter model scenarios. Under wetter scenarios, MNWR could consider not choosing to replace some infrastructure (culverts, pipes, or water control structures) for frequently flooded areas, to reduce financial impacts from flood damages. Under both wet and dry scenarios, better quantifying water use needs can help MNWR better plan for moderately dry years by developing a habitat management plan that prioritizes habitats by wildlife value and water use requirements, and quantifying irrigation needs to predict how changes in CWD will translate to additional water needed for prioritized habitats.

### Use of Hydroclimate and Qualitative Models in Climate Adaptation Planning

The BCM outputs proved very useful for interpreting changes in temperature, precipitation, and event frequency to the extent, magnitude, frequency, and timing of hydrologic changes that impact water supply and security of a refuge with complex water management. However, we demonstrated that a greater value can be applied to model interpretation by incorporating results of the BCM model into a management framework. Development of conceptual models portraying refuge sensitivity permitted a better use of the BCM outputs, by allowing them to be used to improve our understanding of vulnerability of the refuge.

Establishment of tolerance thresholds is important for looking at the implications of different climate-change scenarios on water management. However, this information is often lacking in water management systems, such as the refuge in this case study that have historically relied on professional judgment and qualitative assessment of the response of desired outcomes to variable water supply. By use of water year types defined by management outcomes, we established a process that can be used to better interpret hydroclimate model results into context for land managers. However, caution should be taken because future forecasts presented in this study assume static water management objectives and do not incorporate additional variables such as observed increases in CWD.

Substantial uncertainty was observed between the climate models, as was evidenced by divergent trends in precipitation. However, the development of a framework to place these model outputs into distinct scenarios created an opportunity to look at the range of possible best-case and worst-case scenarios, and identify consensus among model scenarios. The conceptual framework approach used in this study demonstrated that uncertain information can be useful for development of refuge management adaptation strategies.

## Electronic Supplementary Material

Below is the link to the electronic supplementary material.

Supplementary material 1 (XLSX 24 kb)

Supplementary material 2 (XLSX 10 kb)

Supplementary material 3 (XLSX 85 kb)

Supplementary material 4 (XLSX 10 kb)
